# Pulmonary artery intimal sarcoma mimicking pulmonary thromboembolism: A case report

**DOI:** 10.1097/MD.0000000000024699

**Published:** 2021-02-12

**Authors:** Ying-Chun Li, Le-Yao Li, Hai-Chao Tong, Hong-Tao Xu, Shuang Ma, Lian-He Yang, Wan-Lin Zhang, Gina Sotolongo, Endi Wang

**Affiliations:** a Department of Pathology, First Affiliated Hospital and College of Basic Medical Sciences, China Medical University; b Department of Neurology, Sheng Jing Hospital of China Medical University, Shenyang, Liaoning; c Department of Pathology, Hebei Petro China Central Hospital, Langfang, Hebei, China; d Department of Pathology, Duke University Medical Center, Durham, NC.

**Keywords:** differential diagnosis, fibrosarcoma, morphology, pathology, pulmonary artery

## Abstract

**Rationale:**

Pulmonary artery intimal sarcoma is a rare tumor with exceptionally high mortality and easily misdiagnosed as pulmonary thromboembolism pulmonary thromboembolism (PTE) due to the nonspecific clinical presentation and symptom. Misdiagnosis or untimely diagnosis makes the disease progress to an advanced stage and eventually leads to a poor prognosis.

**Patient concerns:**

A 37-year-old Chinese female presented with chest tightness and dyspnea for 3 months. Echocardiography and chest computed tomography revealed an intraluminal obstruction of the pulmonary arteries. Tests of serum tumor makers showed slight elevation for carbohydrate antigen-125, and α-fetoprotein. PTE was suspected according to the radiological and laboratory findings.

**Diagnosis:**

Microscopic findings of the presumed thrombus showed prominent myxoid and edematous background with atypical spindled cells and curvilinear vascularity. Immunohistochemical staining demonstrated that the atypical spindled cells were positive for vimentin but negative for CK, S100, SMA, desmin, CD68, STAT6, CD34, β-catenin, ALK-p80, p53, and MDM2. According to the radiological and pathological findings, the diagnosis of fibrosarcoma of pulmonary artery was made.

**Interventions:**

The patient underwent surgical resection and the mass was excised as completely as possible.

**Outcome:**

Follow-up information showed no evidence of recurrence or metastasis after 3 months postresection.

**Lessons:**

Because of the low incidence rate, nonspecific clinical symptoms, and radiological findings, primary fibrosarcoma of the pulmonary artery is commonly misdiagnosed as PTE. Pathological examination is necessary to confirm the diagnosis.

## 1 Introduction

Pulmonary artery intimal sarcoma (PAIS) is an exceedingly rare malignancy with poor oncological outcomes. It was first described in 1923 by Mandelstamm via autopsy.^[1]^ Subsequently, there were several reports about primary sarcomas arising from major blood vessels, such as the aorta, carotids, inferior vena cava, axillary, femoral, and popliteal arteries, with the pathological typing divided into leiomyosarcomas and fibrosarcomas. In 1960, Wolf et al^[2]^ reported the first English case fibrosarcoma involving the pulmonary trunk. Because of the rareness and nonspecific clinical manifestations and similar imaging findings, pulmonary artery (PA) sarcoma is commonly misdiagnosed as pulmonary thromboembolism (PTE) and clinician wrongly perform antithrombotic and thrombolytic therapies, which induce the development of PAIS. About 42.3% of PAIS patients were misdiagnosed as PTE and could not receive timely and effective treatment.^[3]^ Herein, we report a 37-year-old Chinese female wrongly diagnosed as PAIS initially and was subsequently diagnosed as PTE.

## 2 Case presentation

**Figure 1:**
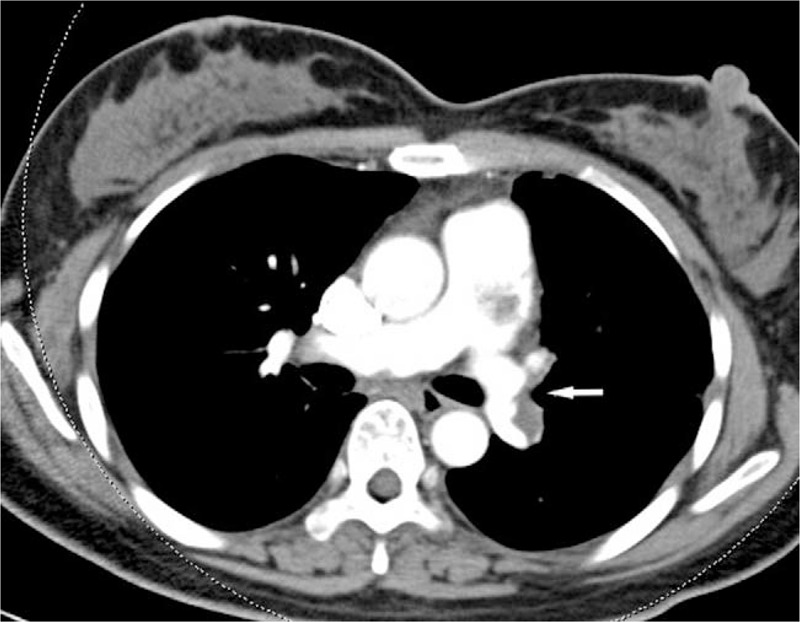
Enhanced chest computed tomography shows an irregular low-density soft tissue lesion (the filling defect is highlighted with white arrow) in the main pulmonary artery and left pulmonary artery.

A 37-year-old Chinese woman came to our hospital complaining of exertional chest discomfort and dyspnea on exertion for 6 months. The patient denied any significant past medical history. On admission, physical examination was unremarkable. Two-dimensional echocardiography revealed lesions in the pulmonary arteries combined with moderate pulmonary hypertension and right ventricular dysfunction. Subsequently, contrast-enhanced chest computed tomography (CT) demonstrated multiple irregular low-density soft tissue lesions in the main pulmonary artery (PA) trunk with perfusion defects in the bilateral PA branches (Fig. 1). Following serum tumor makers were tested, including carbohydrate antigen-125 (CA-125):61.87 μl/ml (0.00–35.00 μl/ml), α-fetoprotein (AFP):9.40 ng/ml (0.00–7.00 ng/ml), carcinoembryonic antigen (CEA):1.61 ng/ml (0.00–4.30 ng/ml), human chorionic gonadotrophin:1.88 mIU/ml (0.00–3.00 mIU/ml). Color Doppler ultrasound of both lower extremities showed no specific changes in the vessels. According to these findings, PTE was suspected, but primary or metastatic malignancy could not be completely excluded. The patient refused (18) F fluorodeoxyglucose positron emission tomography/computed tomography [(18) F-FDG PET/CT]. Median sternotomy and pericardiotomy were performed demonstrating moderate pericardial effusion and dilated cardiomyopathy. Upon incision of the PA, a luminal yellow mass with jelly-like texture was identified; the mass was adherent to the pulmonary trunk intima and extended into the bilateral main pulmonary arterial. Although no capsule was identified, the surgeon resected the mass as completely as possible. At this time, extracorporeal membrane oxygenation was applied to improve the declining oxygen saturation and blood pressure, which could delay the progression of right heart failure due to pulmonary hypertension. Small dose of vasodilator was also provided to support the adjuvant circulation.

**Figure 2:**
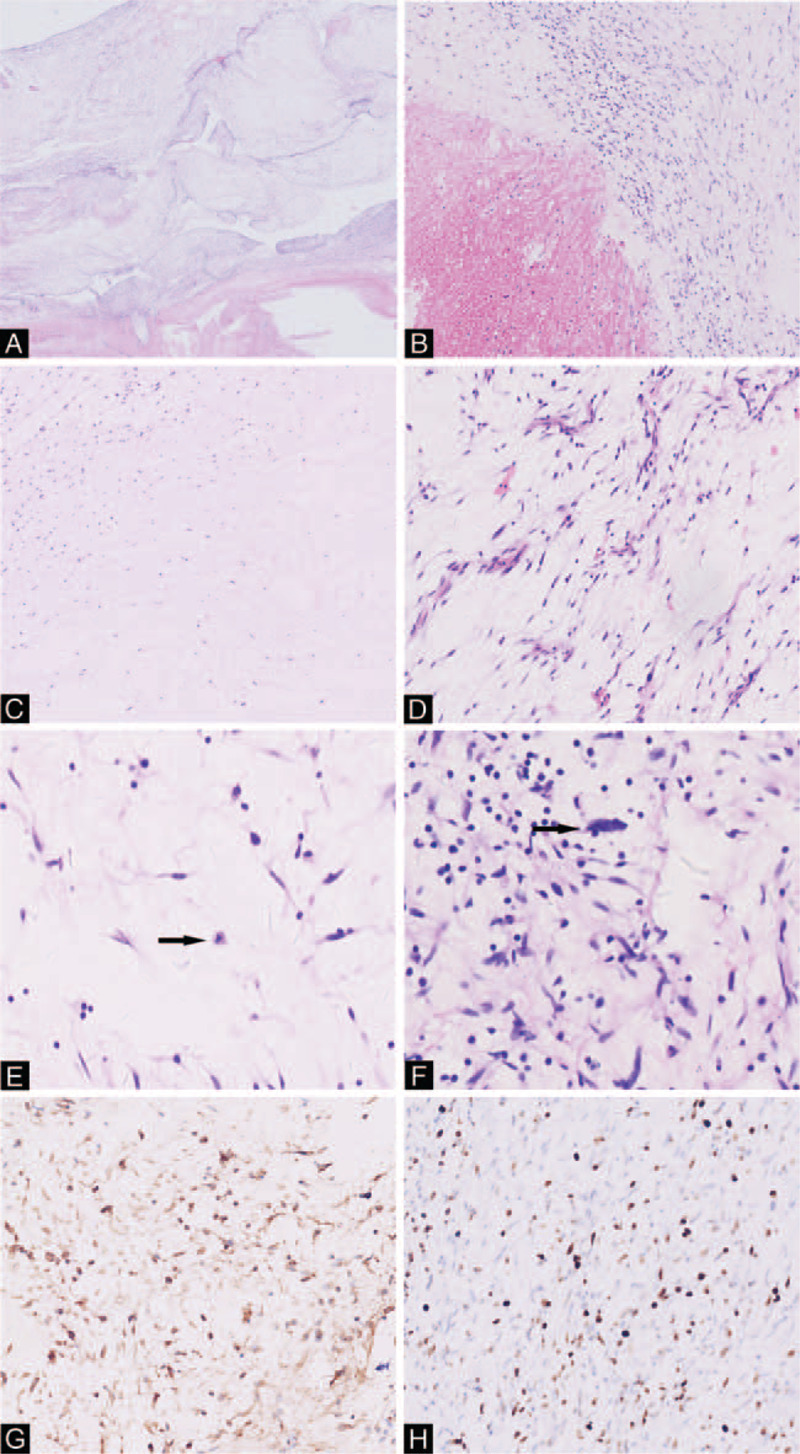
A: The tumor present is an intravascular mass which is closely connected to the vascular intima (4X); B: The tumor is connected with thrombosis (4X); C: Necrosis could be found (4X); D: The spindle tumor cells scattered in the myxoid and edematous background (10X); E: Mitosis could be found occasionally (20X, highlighted with black arrow); F: Rarely, the tumor cells showed great atypia (20X, highlighted with black arrow); G: The tumor cells showed positive expression of vimentin; H: The Ki67 index is about 20% in the hot spot.

Pathological gross examination demonstrated a multinodular mass that was adherent to the vessel wall. Microscopic examination revealed the mass was composed of a hypercellular spindle cell population with mild atypia and increased mitotic activity in a myxomatous and edematous background (Fig. 2). The tumor cells were seen infiltrating the vascular intima, resulting in secondary thrombus formation. Immunohistochemical staining showed the tumor cells were positive for vimentin, but negative for CK, S100, SMA, desmin, myogenin, CD68, STAT6, CD34, CD31, β-catenin, ALK-p80, P53, and MDM2; Ki67 is about 20% in areas of highest activity (Fig. 2). These findings confirm the diagnosis of primary fibrosarcoma of the PA. After the surgery, the patient was treated with prophylactic radiotherapy with a dose of 45 to 56 Gy, 1.8 to 2 Gy each time, once a day, 5 times a week. The postoperative CT showed no clear space-occupying lesions in the PA and its branches, and the follow-up image after 3 months showed no occurrence of the mass.

## 3 Discussion

PAIS is a rare malignancy with an incidence of 0.001% to 0.03%^[4,5]^ with only around 400 cases reported in the literature worldwide.^[6,7]^ Epidemiological studies show that PAIS typically affects middle-aged adults (mean age of 48-years-old) with a slight female predominance. PAIS typically remains a localized mass with slight metastatic ability.^[8]^ Based on the location, it could be divided into mural and intimal sarcoma. For mural sarcoma, the most common pathological type is classic leiomyosarcoma, which develops along the medial smooth muscle of large veins, primarily the vena cava.

Alternatively, intimal sarcoma commonly arises from intimal layer of large blood vessels, such as pulmonary arteries and the aorta.^[4]^ Such sarcomas are characterized by an endoluminal growth that progresses to vessel obstruction or embolic seeding of distal sites.^[9,10]^

The clinical symptoms of PAIS include dyspnea, cough, hemoptysis, or chest pain which shares a clinical presentation with respiratory infection, heart failure, and pulmonary embolism.^[11,12]^ Because the clinical presentation of PAIS is nonspecific, the diagnosis of PAIS is particularly challenging, especially to inexperienced clinicians. Among all the differential diagnoses, it is imperative that clinicians rule out life-threatening pulmonary emboli. However, unlike the acute nature of pulmonary emboli, the symptoms of PAIS are more gradual in presentation. In our case, the patient presented with a 6-month history of progressing dyspnea on exertion, which favors the diagnosis of PAIS. Additional findings of PAIS include fever, cachexia, inflammatory anemia, and increased erythrocyte sedimentation rate also indicate the diagnosis of PAIS, especially if the patient denies any risk factors for deep venous thrombosis.^[12]^ Little is known about how serum tumor markers can be used in the diagnosis of PAIS. Interestingly, our case showed slight evaluated level of CA-125, AFP, and CEA which indicates a tumor lesion. However, it is unclear at this point whether CA-125, AFP, or CEA could serve as the biomarkers of PAIS and more investigation is needed.

Radiological study is a useful tool in the diagnosis of PAIS and the combination of chest CT-angiography and cardiac ultrasound is helpful for ruling out PTE.^[13,14]^ Typically, chest contrast-enhanced CT of PAIS demonstrates a heterogeneous, irregular low-density mass with filling defect of the pulmonary vasculature and vascular distention.^[13,15]^ In addition to PA space-occupying lesions in chest CT, multiple pulmonary nodules or hilar tumors are usually helpful for the diagnosis of PAIS rather than PTE, which is usually accompanied by wedge-shaped pulmonary infarction.^[16–18]^ In CT pulmonary angiography, PAIS mainly occurs in the pulmonary trunk, and could extend proximally to the pulmonary valve and even right ventricular outflow tract, extend distally to the left and right pulmonary arteries and branches. The proximal end of the mass in PAIS is mostly swollen, bulging or lobulated.^[19–21]^ Hui-Li Gan et al^[22]^ suggested that the “wall eclipsing sign” usually occurred in PAIS, where low-density mass almost occupies the main PA and the proximal parts and distal ends as well as erodes any of pulmonary arteries. However, PTE mostly involves the left or right PA, with saddle-shaped lump on imaging, and there is no finding of “wall eclipsing sign.”^[3,22]^ In addition, PAIS has increased heterogeneity compared to PTE in CT pulmonary angiography.^[23]^ On magnetic resonance imaging, The T2W signal intensity of PAIS is higher than that of PTE.^[24]^ Some researchers recently suggested that (18) F-FDG PET/CT could be useful in distinguishing PAIS from PTE based on the maximum of standard uptake value, which mostly revealed hypermetabolism in tumors,^[16,17]^ but PAIS cannot not be ruled out when there is no obvious abnormality in (18) F-FDG PET/CT.^[25]^ In our case, the contrast-enhanced CT demonstrated multiple irregular low-density soft tissue lesions in the main PA trunk with perfusion defects in bilateral PA branches, which is consistent with previous reports.

Macroscopically, PAIS is always an intravascular mass which is closely adherent to the vascular intima, and can extend down into adjacent vessels.^[4,26]^ Histologically, the tumor is commonly composed of spindle or epithelioid cells with various atypia; myxoid and fibrosis changes are common, as noted in our case.^[4,27]^ Immunohistochemical staining for PAIS shows variable expression of desmin and smooth muscle actin but negative for epithelial markers, factor VIII, CD31, CD34, and S100. It has been reported that frequent MDM2 expression could be detected in PAIS because of the high rate of amplification of MDM2 (65%), but our case showed negative expression of MDM2. Nowadays, fluorescent in-situ hybridization examination shows frequent amplification of PDGFRA (81%), MDM2 (65%), and EGFR (76%), which can be used to improve diagnostic identification of PAIS.^[28]^

Surgery is the main treatment of PAIS. It has been reported that the patients who undergo curative resection have a longer overall survival than those who undergo incomplete resection (median overall survival of 36.5 vs 11 months, respectively).^[12]^ Postoperative adjuvant chemotherapy and radiotherapy, however, do not demonstrate statistically significant improved survival.^[29]^

In conclusion, PAIS is a rare disease which could be easily misdiagnosed due to the nonspecific indolent clinical presentation and its symptomatic similarity to PTE. It can be diagnosed preoperatively via the use of multiple radiological modalities; and postoperatively with a combination of histopathological examination, immunohistochemical staining, and fluorescent in-situ hybridization studies.

## Author contributions

**Conceptualization:** Hai-Chao Tong, Wan-Lin Zhang.

**Data curation:** Ying-Chun Li.

**Formal analysis:** Hai-Chao Tong, Shuang Ma.

**Funding acquisition:** Shuang Ma, Lian-He Yang.

**Investigation:** Ying-Chun Li, Shuang Ma.

**Methodology:** Hong-Tao Xu.

**Project administration:** Lian-He Yang.

**Resources:** Le-Yao Li.

**Software:** Le-Yao Li.

**Supervision:** Endi Wang.

**Validation:** Hong-Tao Xu.

**Visualization:** Wan-Lin Zhang.

**Writing – original draft:** Ying-Chun Li.

**Writing – review & editing:** Lian-He Yang, Gina Sotolongo.

## Abbreviations

Abbreviations: (18) F-FDG PET/CT = (18) F fluorodeoxyglucose positron emission tomography/computed tomography, AFP = α-fetoprotein, CA-125 = carbohydrate antigen-125, CEA = carcinoembryonic antigen, CT = computed tomography, PA = pulmonary artery, PAIS = pulmonary artery intimal sarcoma, PTE = pulmonary thromboembolism.
